# Treatment of Acute Lymphoid Leukemia Refractory to Classic First-Line and Rescue Protocols

**Published:** 2020-04-01

**Authors:** Flávia Tobaldini Russo, Maria Cristina Martins de Almeida Macedo, Pedro Amoedo Fernades, Larissa Yukari Okada, Lucas Augusto Monetta da Silva, Camila Menin Simões, Jamilla Neves Cavalcante, Aline de Almeida Simões, Manuella de Souza Sampaio Almeida, Maricy Almeida Viol Ferreira Lopes, Roberto Luiz da Silva

**Affiliations:** 1 Instituto Brasileiro de Controle do Câncer (IBCC), São Paulo, Brazil; 2 Bio Sana`s serviços Médicos, São Paulo, Brazil; 3 Faculdade de Ciências Médicas Santa Casa de São Paulo, São Paulo, Brazil

**Keywords:** Acute lymphoblastic leukemia, Lymphoid cells, Relapsed leukemia

## Abstract

Acute Lymphoblastic Leukemia is a very aggressive malignant disorder of lymphoid cells in adults, with recurrence (30 to 60% of the cases) after the initial treatment. Until this moment, there is no gold standard therapy for the treatment of adult patients with acute relapsed/refractory lymphoblastic leukemia. In this case report, we describe two cases of relapsed leukemia: one of lymphocytic leukemia B and one of trilineage leukemia, which presented a satisfactory response to treatment with Bortezomib associated with Vincristine, Dexamethasone, and Bendamustine.

## Introduction

 Acute Lymphoblastic Leukemia (ALL) is defined as a malignant disorder of lymphoid cells that leads to this cells proliferation in the bone marrow and extra spinal bone^   1 ^.

Contrary to what is observed in the pediatric setting, ALL in adults is a very aggressive disease in which response to treatment is still far below expected. Although rates of de novo ALL remission in adults after induction regimen with first-line chemotherapy regimens are around 80%, disease-free survival progression is still estimated in only 40% of patients^   2 ^.

Besides, it is known that 30 to 60% of these patients who respond to the initial treatment will present neoplasia recurrence, despite the consolidation and maintenance therapies, which include chemotherapy and allogeneic bone marrow transplantation^   3 ^.

To date, there is no gold standard therapy for the treatment of adults with acute relapsed/refractory lymphoblastic leukemia. We have reported here two cases of relapsed leukemia, one of lymphocytic leukemia B and one of trilineage leukemia, which presented a satisfactory response to treatment with Bortezomib associated with Vincristine, Dexamethasone, and Bendamustine. 

## Case presentation

**Case 1**

A 31-year-old male patient was diagnosed in another service with non-Hodgkin's lymphoma without other specifications (WOS) in December 2016. At diagnosis, there was no evidence of spinal cord involvement due to neoplasia. He underwent standard treatment with CHOEP (Cyclophosphamide, Doxorubicin, Vincristine, Etoposide, and Prednisone) for six unresponsive cycles, progressing the disease one month after the end of treatment.

After progression of the disease, the patient underwent DHAP rescue treatment (Cytarabine, Cisplatin, and Dexamethasone) for three cycles, followed by autologous hematopoietic stem cell transplantation in November 2017.

Six months after autologous bone marrow transplantation, the patient was diagnosed with trilineage leukemia, and the bone marrow immunophenotyping was composed of positive anomalous cells for cytoplasmic CD3, CD7, CD19, CD79a, myeloperoxidase (MPO) and nuclear terminal deoxynucleotidyl transferase (TdT).

Patient was treated with Hyper-CVAD regimen, which includes in the odd cycles: Cyclophosphamide, Vincristine, Doxorubicin, Dexamethasone, and in the even cycles: Methotrexate in high doses, Cytarabine and Methylprednisolone. Since he did not respond, he was submitted to the IDAFLAG scheme, which was composed of Idarubicin, Fludarabine, Cytarabine, but also did not respond to the protocol, being considered refractory and referred to our service.

His myelogram of 10/31/18 showed 60% of anomalous blasts with positivity for the same markers that were positive at diagnosis.

An alternative protocol for refractory ALL, which included Bortezomib at a dose of 1.3 mg/m2 on days 1, 4, 8 and 11, Vincristine at a dose of 1.5 mg/m2 (maximum dose 2 mg) on days 1, 8, 15 and 22, Dexamethasone at a dose of 40 mg/day from day 1 to days 4, day 8 to 11 and day 15 to 18 and Bendamustine at 120 mg / m2 on days 1 and 2 was considered.

After two cycles of the aforementioned treatment, on 12/28/18, the patient was in cytomorphological remission by the myelogram and the bone marrow immunophenotyping showed only 1.7% of anomalous cells with immunophenotype similar to that of the diagnosis being considered as positive for minimum residual disease (MRD).

The patient was submitted to allogeneic related transplantation with a full match female donor in January 2019. The conditioning was made with Cyclophosphamide at a dose of 60 mg/kg for 2 days and total body irradiation at a dose of 12 Gy for 3 days.

The cell source chosen was peripheral stem cells. The patient developed grade I graft-versus- host disease in the gastrointestinal tract, responsive to corticosteroids.

Currently, the patient is at approximately day+160 of the transplant, in an outpatient medical follow-up, with no signs of chronic graft- versus- host disease, cytomorphological remission, MRD and complete chimerism until the last medullary evaluation.

**Case 2**

A 31-year-old male patient was diagnosed in November 2017 with acute B-cell lymphoblastic leukemia, negative Ph, presenting a diagnosis of unchanged bone marrow karyotype (46 XY) and FISH (Fluorescence *in situ* hybridization) with IGH gene translocation and alteration of RUNX1.

Given the diagnosis, the patient was treated with GRAAL protocol. Medullary evaluation performed on the eighth day of the protocol evidenced 5.46% of blasts in immunophenotyping and was then directed and classified as part of the high risk protocol group.

At the end of the induction, the patient was in cytomorphological remission; however, with 0.15% of anomalous lymphoid blasts in immunophenotyping being considered as positive MRD, which was maintained until the end of the consolidation phase.

Due to the persistence of positive MRD after consolidation, it was decided to start therapy with Blinatumomabe in February 2018; however, the patient evolved unfavorably with loss of haematological response and higher blast growth during therapy.

Rescue therapy with FLAG-Ida protocol was initiated on 03/04/18; however, a 14% of blasts was shown in the myelogram collected during recovery, leading the patient to be referred to our service.

A new medullary evaluation was performed on 05/23/19 in which the immunophenotyping showed 32% of blasts positive for cytoplasmic CD79a, CD19, CD34, nuclear TdT, CD10, CD22 and negative for MPO, CD3, CD7 and CD13 being compatible with common B-cell acute lymphoid leukemia.

Initiated on 05/27/19 the first course of treatment including Bortezomib, Vincristine, Dexamethasone and Bendamustine in the same dosages described in the above report. Myelogram was collected for evaluation of response on 06/29/18 that showed complete cytomorphological response (0.8% of blasts) and immunophenotyping showing 0.5% of blast cells with the same profile of diagnostic markers being considered as positive MRD.

Since the patient had a compatible donor available, he was submitted to a related allogeneic bone marrow transplant in July 2018 after conditioning with Cyclophosphamide at a dose of 60 mg/kg for 2 days and total body irradiation at a dose of 12 Gy divided in 3 days.

He remained in complete remission for 5 months; however, in December 2018, the patient presented a new progression of the disease. He was again treated with the aforementioned protocol associated with Blinatumumab. The patient obtained a satisfactory medullar response. He was on a second allogeneic bone marrow transplant schedule, but he died on 06/02/19 because of a systemic fungal infection that was not responsive to the treatment.

## Discussion

 For being a serious disease and with high rates of relapse and refractoriness, new drugs for ALL are constantly being studied, as well as the resistance mechanisms of neoplastic cells to chemotherapeutics. Bortezomib, the first proteasome inhibitor released by the Food and Drug Administration (FDA) for multiple myeloma and relapsed non-Hodgkin's lymphoma, has shown activity in ALL. One of its main roles is the inhibition of the aforementioned NK kappa pathway^   4 ^. In the treatment of relapsed/refractory ALL, Bortezomib as monotherapy did not show very satisfactory responses. However, its association with other drugs such as Vincristine, Dexamethasone, Asparaginase, and Doxorubicin potentiates the effect of the latter, increasing the efficacy of the treatment of children^   4 ^.

Also, in the setting of adults with relapsed/refractory ALL, the combination of Bortezomib and classical regimens showed satisfactory responses^5^^, ^^6^. Zhao et al. (2015)^   7 ^used Bortezomib in combination with first-line drugs in nine adults with relapsed/refractory lymphoid leukemia as a bridge for hematopoietic stem cell transplantation. Of the nine patients evaluated, eight achieved complete remission before bone marrow transplantation and the treatment-related toxicities were well tolerated^   7 ^. Bendamustine is an alkylating agent that induces DNA damage leading to cell apoptosis that has already had its benefits well described in chronic lymphocytic leukemia and recurrent and refractory non-Hodgkins lymphomas^   4 ^. In the context of new drugs, Bendamustine was used in children as monotherapy for treatment of acute relapsed/refractory leukemias of both myeloid as lymphoid lineages with satisfactory response^        8 ^.

Approximately 90% of the B- lymphoid precursor cells express the CD19 surface antigen. From this, Blinatomumab, a bispecific antibody that binds both CD3 + cytotoxic T cells as CD19 + B cells, has recently been developed. In this way, the medication allows the patient's endogenous T cells to recognize and eliminate CD19 + B blasts^   9 ^.

The efficacy of Blinatumumabe has already been shown in patients with relapsed/refractory ALL as well as in minimal residual disease in patients who were in cytomorphologic remission^9^^,^^10^. Currently, manipulation of T cells to express a chimeric antigen receptor (CAR-T) has emerged as a promising immunotherapy in the treatment of refractory or relapsing oncohematologic diseases. In ALL, CAR-T therapy directed to CD19 showed excellent response rates of around 70-90%^11^^-^^13^. The remission duration of patients submitted to CAR-T treatment still requires more robust studies and a longer follow-up time^   14 ^.

Despite the apparent efficacy shown by the studies, access to CAR-T therapy is still very restricted and extremely costly, which makes it difficult to implement in the vast majority of services in Brazil and others Latin American countries.

## CONCLUSION

 Effective treatment of patients with acute relapsed/refractory lymphoid leukemia is still a challenge and is a subject of great interest and much studies in current time. The availability of effective and well-tolerated therapies is of paramount importance to ensure increased progression-free survival and overall survival of these patients. The cases reported in this study show the viability in the treatment of relapsed/refractory ALL using economically accessible medications and the efficacy of protocols used as an alternative to allogeneic bone marrow transplantation with reduced costs as compared to schemes with CAR-T cells. However, there is a need for prospective studies to prove the efficacy of this protocol for recurrent and refractory ALL. From these case reports, the Hematology team of our service proposed a prospective study in an attempt to offer a rescue treatment for recurrent and refractory ALL, which has already exhausted the conventional therapeutic possibilities with drugs that are accessible to our service.

## References

[1] Pui CH, Robison LL, Look AT (2008). Acute lymphoblastic leukaemia. Lancet.

[2] Fielding AK, Richards SM, Chopra R (2007). Outcome of 609 adults after relapse of acute lymphoblastic leukemia (ALL); an MRC UKALL12/ECOG 2993 study. Blood.

[3] Frey NV, Luger SM (2015). How I treat adults with relapsed or refractory Philadelphia chromosome–negative acute lymphoblastic leukemia. Blood.

[4] Messinger YH, Gaynon PS, Sposto R (2012). Bortezomib with chemotherapy is highly active in advanced B-precursor acute lymphoblastic leukemia: Therapeutic Advances in Childhood Leukemia &amp; Lymphoma (TACL) Study. Blood.

[5] Dewar R, Chen ST, Yeckes-Rodin H (2011). Bortezomib treatment causes remission in a Ph+ALL patient and reveals FoxO as a theranostic marker. Cancer Biol Ther.

[6] Hu X, Xu J, Sun A (2011). Successful T-cell acute lymphoblastic leukemia treatment with proteasome inhibitor bortezomib based on evaluation of nuclear factor-κB activity. Leuk Lymphoma.

[7] Zhao J, Wang C, Song Y (2015). Treatment of refractory/relapsed adult acute lymphoblastic leukemia with bortezomib-based chemotherapy. Int J Gen Med.

[8] Fraser C, Brown P, Megason G (2014). Open-label bendamustine monotherapy for pediatric patients with relapsed or refractory acute leukemia: efficacy and tolerability. J Pediatr Hematol Oncol.

[9] Kantarjian H, Stein A, Gökbuget N (2017). Blinatumomab versus Chemotherapy for Advanced Acute Lymphoblastic Leukemia. N Engl J Med.

[10] Gökbuget N, Dombret H, Bonifacio M (2018). Blinatumomab for minimal residual disease in adults with B-cell precursor acute lymphoblastic leukemia. Blood.

[11] Davila ML, Riviere I, Wang X (2014). Efficacy and toxicity management of 19-28z CAR T cell therapy in B cell acute lymphoblastic leukemia. Sci Transl Med.

[12] Maude SL, Frey N, Shaw PA (2014). Chimeric antigen receptor T cells for sustained remissions in leukemia. N Engl J Med.

[13] Lee DW, Kochenderfer JN, Stetler-Stevenson M (2015). T cells expressing CD19 chimeric antigen receptors for acute lymphoblastic leukaemia in children and young adults: a phase 1 dose-escalation trial. Lancet.

[14] Maude SL, Teachey DT, Porter DL (2015). CD19-targeted chimeric antigen receptor T-cell therapy for acute lymphoblastic leukemia. Blood.

